# Successful defibrillation verification in subcutaneous implantable cardioverter‐defibrillator recipients by low‐energy shocks

**DOI:** 10.1002/clc.23184

**Published:** 2019-04-25

**Authors:** Mauro Biffi, Matteo Ziacchi, Andrea Angeletti, Andrea Castelli, Giulia Massaro, Cristian Martignani, Mariolina Lovecchio, Sergio Valsecchi, Igor Diemberger

**Affiliations:** ^1^ PoloCardio‐Toraco‐Vascolare Azienda Ospedaliero‐Universitaria di Bologna Bologna Italy; ^2^ Università di Bologna Bologna Italy; ^3^ Boston Scientific Italy Milano Italy

**Keywords:** defibrillation test, energy, implantable defibrillator, subcutaneous, ventricular fibrillation

## Abstract

**Background:**

The subcutaneous implantable cardioverter‐defibrillator (S‐ICD) is an effective alternative to the transvenous one. Defibrillation efficacy depends on maximum device output and on the optimal device location at device implantation.

**Hypothesis:**

We sought to investigate the defibrillation safety margin in real life clinical practice.

**Methods:**

We sought to understand what is the efficacy of induced ventricular fibrillation (VF) termination at S‐ICD implantation using lower energies than the recommended 65 J.

**Results:**

Sixty‐four consecutive S‐ICD recipients underwent VF termination attempts at implantation with energies ranging from 20 to 50 J. Overall, VF termination occurred in 84% of patients with ≤40 J, in 88% with 45 J, and in 100% with 60 J. Intermuscular S‐ICD placement was associated with 94% VF termination at ≤40 J. An ejection fraction <35% was associated to higher energy requirement for defibrillation; however, an intermuscular S‐ICD placement conferred 90% defibrillation efficacy at 31 ± 5 J in this patients subset.

**Conclusions:**

This is a hypothesis‐generating observation that prompts a methodologically correct investigation to prove that a 60 J output S‐ICD can provide an adequate safety margin to terminate VF in clinical practice. This would enable superior device longevity and/or device downsizing for pediatric/small size patients.

## 1 INTRODUCTION

The practice of defibrillation threshold testing (DFT) or of defibrillation verification at the time of transvenous implantable cardioverter‐defibrillators (ICDs) implant has decreased along years, based on similar patients' outcome irrespectively of DFT testing.^1^ Contemporary recommendations for defibrillation verification focus on selected populations and on atypical implant configurations.^2^

The recently developed subcutaneous ICD (S‐ICD) is an effective alternative to transvenous ICD that does not require an endovascular lead placement.^3^, ^4^ However, studies on the safety of DFT avoidance are still lacking with S‐ICD, as well as studies investigating the factors potentially associated with higher DFTs. For this reason, functional defibrillation testing is still recommended at S‐ICD implantation.^2^ Current S‐ICD devices deliver a maximum of 80 J, thus the test is usually conducted by delivering a shock energy of 65 J to ensure a safety defibrillation margin of at least 15 J. Recent findings from clinical practice in the US and Europe^5^, ^6^, ^7^, ^8^ show high rates (above 90%) of successful conversion at ≤65 J, but limited data on the conversion success at lower energies exist.

The aim of this study was to describe our experience of ventricular fibrillation (VF) termination with lower energy S‐ICD shocks, and to identify factors potentially associated with test failure.

## 2 METHODS

### 2.1 Study design

**Figure 1:**
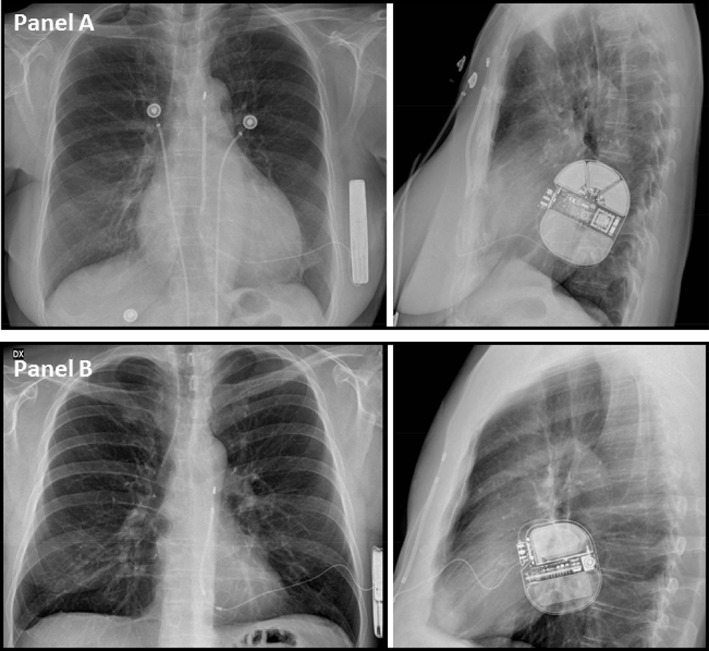
Antero‐posterior and left‐lateral view of an intermuscular (Panel A) and a subcutaneous (Panel b) S‐ICD. VF termination occurred at 20 J (A) and 30 J (B), respectively. S‐ICD, subcutaneous implantable cardioverter‐defibrillator; VF, ventricular fibrillation

We included in this analysis all consecutive patients undergoing implantation of an S‐ICD (Boston Scientific Inc., Natick, Massachusetts) from February 2015 to October 2018 at our Institution. The Institutional Review Board approved the study, and all patients provided written informed consent for data storage and analysis. Baseline assessment comprised the collection of demographic data and medical history, clinical examination, 12‐lead electrocardiogram, echocardiographic evaluation, magnetic resonance scanning, and coronary angiography (when clinically indicated). An adequate S‐ICD sensing was verified before implantation by the surface electrocardiogram (ECG) screening method that is based on a dedicated ECG morphology tool.^9^ Device surgery was undertaken under local anesthesia with ropivacaine, plus sedation with propofol (1 mg/kg bolus + infusion as to maintain spontaneous breathing). Along time, we moved from a subcutaneous implant of the defibrillator can (placed posterior to the mid‐axillary line) to an intermuscular placement under the latissimus dorsi that enables a more posterior location and a smaller distance from the chest wall (Figure 1). The intermuscular placement is achieved by skin incision at the axillary midline: the anterior insertion of the latus dorsi is located by dissecting the subcutaneous plane parallel to the rib course; then separation of the muscle layers is easily achieved by blunt dissection, and a posterior pocket between the latus dorsi and the intercostal plane is created (scapula inferior angle is felt when sizing the pocket by fingers). The intermuscular device placement is more posterior than the subcutaneous one, being located posteriorly to the posterior axillary line, and enables a close contact of the device can with the intercostal plane. Thus, the totality of the ventricular mass is included in the defibrillation vector. Defibrillation verification of induced VF (50 Hz transthoracic pacing) occurred as per the manufacturer's recommendation to ensure a 15 J safety defibrillation margin. For the sake of increased safety, we explored higher safety margins by delivering the first shock energy ≤50 J since our earliest procedures. As the confidence in the system increased, we lowered the first‐attempt energy to understand the defibrillation efficacy in a real‐life unselected population of S‐ICD recipients. In case of failure, the second trial at defibrillation verification used a higher energy, in any case ≤60 J. In the event of a second failure at higher energy, reverse polarity at the secondly tested energy was used. There was no systematic approach at the choice of the first attempt delivered energy based on specific patients' characteristics such as body mass index (BMI) or ejection fraction.

### 2.2 Statistical analysis

Descriptive statistics are reported as means ± SD. Categorical variables are reported as percentages. Differences between mean data were compared by means of a *t* test for Gaussian variables, and by the Mann‐Whitney non‐parametric test for non‐Gaussian variables. Differences in proportions were compared by means of χ^2^ analysis or Fisher's exact test, as appropriate. Logistic regression analysis was used to determine the association between successful conversion at the first shock and clinical characteristics and implantation variables and to estimate the odds ratios and the 95% confidence intervals. A *P* value <.05 was considered significant for all tests. All statistical analyses were performed by means of STATISTICA software, version 7.1 (StatSoft, Inc, Tulsa, Oklahoma).

## 3 RESULTS

### 3.1 Study population and S‐ICD implantation procedure

A total of 72 patients underwent S‐ICD implantation. Table 1 shows the baseline clinical variables in the study population. Patients were predominantly male (74%), relatively young (47 ± 17 years), and only a minority showed severely depressed systolic function (28% with left ventricular ejection fraction ≤35%). The S‐ICD generator was positioned in a standard subcutaneous pocket in 30 (42%) patients, while an inter‐muscular approach was adopted in the remaining patients (Figure 1). In 49 (68%) patients, the S‐ICD generator was located superiorly to the cardiac apical shadow on supine fluoroscopy.

**Table 1:** Demographics and baseline clinical parameters

Parameter	All patients (n = 72)	Intermuscular (n = 42)	Subcutaneous (n = 30)
Male gender, n (%)	53 (74)	31 (74)	22 (73)
Age, years	47 ± 17	45 ± 17	50 ± 16
Body Mass Index, kg/m^2^	26 ± 5	26 ± 5	26 ± 5
Body Surface Area, m^2^	1.9 ± 0.2	1.9 ± 0.2	2.0 ± 0.2
Secondary prevention of SCD, n (%)	22 (31)	11 (26)	11 (37)
Ischemic cardiomyopathy, n (%)	12 (17)	6 (14)	6 (20)
Hypertrophic cardiomyopathy, n (%)	24 (33)	16 (38)	8 (27)
LV ejection fraction, %	52 ± 18	52 ± 18	52 ± 18
LV ejection fraction ≤35%, n (%)	20 (28)	12 (29)	8 (27)
LV end diastolic volume, mL	124 ± 52	118 ± 47	132 ± 70
Maximum LV thickness, cm	1.6 ± 0.8	1.7 ± 0.8	1.4 ± 0.6
LV mass indexed, g/m^2^	153 ± 58	160 ± 60	143 ± 51

Abbreviations: SCD, sudden cardiac death; LV = left ventricular.

### 3.2 Efficacy of VF termination

In 8 (11%) patients, defibrillation verification was not performed for unwillingness of the parents (2 minors) or patient refusal (1 adult patient), clinical instability in 2 heart failure patients, and non‐inducibility of sustained VF in three patients. Of the remaining 64 patients who underwent defibrillation verification, 38 had an intermuscular, and 26 a subcutaneous generator placement; the first conversion attempt occurred at a mean shock energy of 33 ± 7 J with a shock impedance of 77 ± 22 Ohm, respectively at 31 ± 7 J (impedance 75 ± 23 Ohm) in intermuscular implants and at 35 ± 5 J (impedance 82 ± 18 Ohm) in subcutaneous implants, and was successful in 50 patients (78%). A second test, performed at a mean energy of 47 ± 11 J, was successful in the 14 patients in which the first shock did not convert VF.

**Figure 2:**
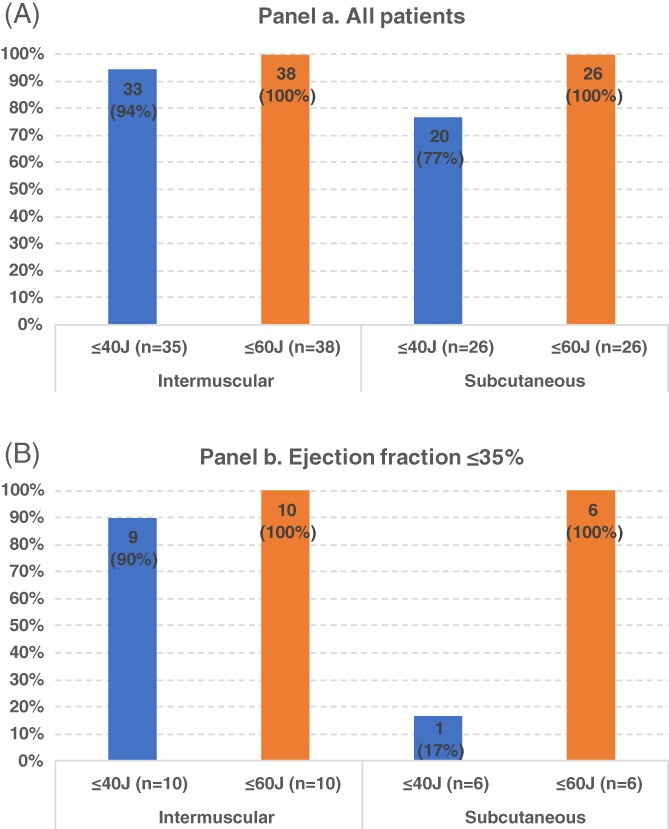
Efficacy of VF termination attempts at implantation according to delivered energy in the overall population (panel A), and in the subgroup with systolic LV dysfunction (panel B). VF, ventricular fibrillation

The first successful attempt was delivered at 31 ± 8 J in intermuscular device recipients, and at 36 ± 5 J in subcutaneous device recipients (*P* = .014). In particular, successful defibrillation was obtained at ≤40 J in 33/35 (94%) intermuscular S‐ICD recipients (Figure 2, panel A), while the remaining three patients had a successful first attempt respectively at 45 J (1 patient) and 50 J (2 patients), and a lower energy was not tested. Successful defibrillation at ≤40 J was obtained in 20/26 (77%, *P* = .063 vs intermuscular) subcutaneous S‐ICD recipients, since 2 patients failed at 30 J, 2 at 35 J, 1 each at 40 J, and 45 J, all being defibrillated with 50 or 60 J at the second trial (Figure 2, panel A).

On logistic regression analysis of clinical characteristics (Table 2), the variables associated with failed VF termination at ≤40 J were higher BMI, left ventricular ejection fraction ≤35% and left ventricular diastolic volume. Among the 16 patients with ejection fraction ≤35% who underwent defibrillation verification, 10 had an intermuscular and 6 a subcutaneous S‐ICD: only 1 of the former 10 failed a first attempt at 40 J, whereas 5 of the latter 6 had a first attempt failure respectively at 30 J (2 patients), 35 J, 40 J, and 45 J (1 patient each; Figure 2, Panel B). The successful conversion attempt was delivered at a mean shock energy of 35 ± 10 J in intermuscular recipients and at 55 ± 3 J in subcutaneous recipients (*P* < .001). A first attempt at a ≥ 40 J energy was more frequently delivered in patients with a BMI ≥ 28, though this behavior was not a pre‐defined systematic approach: 10/14 (71%) of patients with a first attempt at ≥40 J (mean BMI = 28.5 ± 2.8) had a BMI≥28, whereas only 14/50 (28%) patients with a < 40 J first attempt (mean BMI 26.0 ± 4.2) had a BMI ≥28 (*P* = 0.005).

**Table 2:** Univariate analysis of clinical characteristics associated with failed VF termination at ≤40 J

	Univariate analysis		
	OR	95% CI	*P*
Body Mass Index	1.24	1.03‐1.50	.023
Ischemic Cardiomyopathy	3.38	0.67‐17.00	.140
LV ejection fraction ≤35%	12.90	2.26‐73.64	.004
LV end diastolic volume	1.01	1.00‐1.02	.028
Maximum LV thickness	1.20	0.89‐1.62	.233
LV mass indexed	0.99	0.99‐1.01	.929

Abbreviation: LV, left ventricular.

Twenty‐two hypertrophic cardiomyopathy patients with a mean wall thickness of 2.5 ± 0.7 cm underwent defibrillation verification (mean successful shock energy of 35 ± 8 J). Twenty patients were successfully defibrillated at ≤40 J (31 ± 5 J), 1 patient was successfully defibrillated at 60 J after a failed attempt at 30 J, and the remaining patient had a successful first attempt at 50 J. These two latter patients had a subcutaneous placement, a high BMI (27 and 38, respectively), whereas LV mass index (590 and 369 g/m^2^) and maximum thickness (29 and 24 mm) were not significantly different from the other 20 patients (389 ± 141 g/m^2^, 25 ± 7 mm). Six patients with a maximum LV thickness in the range of 30 to 41 mm were successfully defibrillated with ≤30 J.

## 4 DISCUSSION

In our experience of VF termination at the time of S‐ICD implantation, we observed high defibrillation success rates at low energy, suggesting that the safety margin of currently adopted systems is frequently higher than the usually accepted 15 J. Moreover, we found that ejection fraction ≤35% was associated with test failure. Nonetheless, when low ejection fraction patients are considered, a first attempt at ≤40 J was effective in 90% of intermuscular S‐ICD recipients compared with only 16% of subcutaneous recipients.

In current clinical practice, S‐ICD systems are usually tested at 65 J, and high successful rates have been reported in recent literature with this output. In our series, all patients failing a low‐output shock were cardioverted with ≤60 J. In the Evaluation of factors impacting clinical outcome and cost effectiveness of the S‐ICD (EFFORTLESS S‐ICD) Registry,^7^ the proportion of patients showing at least 1 successful conversion test at ≤65 J was 91.6%. In the S‐ICD System Post‐Approval Study,^5^ shock energy of ≤65 J was successful in 91.2% of patients. In the retrospective analysis of S‐ICD implants reported to the National Cardiovascular Data Registry ICD Registry,^6^ 92.7% of patients were successfully defibrillated with a ≤ 65 J shock. In the recently published analysis of Italian clinical practice,^8^ shock energy of ≤65 J was successful in 93.9% of patients. None of the abovementioned studies^5^, ^6^, ^7^, ^8^ specified that VF termination was attempted at <65; only the patients of the initial evaluation of the S‐ICD^10^ underwent full step‐down DFT testing, and the study demonstrated that the system was effective in terminating induced VF with a mean energy of 36.6 J. In our intermuscular S‐ICD recipients, the success rate was ≥90% both in the overall population and in low ejection fraction patients at a mean 31.5 J energy that compares favorably with the study by Bardy. Heist et al.^11^ showed through computer modeling that sub‐coil adipose tissue increased the DFT significantly, as well as a generator anterior positioning. In agreement with these findings, Do et al^12^ reported that in their single‐center experience, the energy required to defibrillate appeared associated with increased BMI and body surface area, making the point that intermuscular placement may confer an advantage in terms of energy requirement for VF termination. Beyond a more posterior can placement (Figure 1), electrode tunneling in the subcutaneous fat along the sternum may occur in obese patients. Similarly, Friedman et al^13^ found an association between increased body mass and lower defibrillation safety margin among S‐ICD recipients. In our study, the average BMI was 26 ± 5Kg/m^2^, quite different from the value of 31 ± 7 kg/m^2^ in the population described by Do et al.^12^ and from the 29 ± 7 kg/m^2^ reported by Friedman et al..^13^ Nonetheless, we confirmed the association between high BMI and defibrillation test failure (Table 2). A thinner body habit enables to place both the subcutaneous coil and the generator directly over the fascia without underlying fat tissue, thus resulting in lowered energy requirement to terminate VF. Moreover, we did not confirm the previously reported association between left ventricle wall thickness and defibrillation success^12^: our study is indeed more powered to assess the role of hypertrophy, because 33% of our patients had hypertrophic cardiomyopathy (the six with wall thickness in the 30‐41 mm range had successful VF termination with ≤30 J), and the mean wall thickness of the whole population was 16 ± 8 mm, well above other literature reports.^3^, ^4^, ^5^, ^6^, ^7^, ^8^, ^12^, ^13^ As described by Friedman et al.,^13^ severely decreased ejection fraction was associated to a lower defibrillation safety margin also in our experience. However, defibrillation was successful at low shock energies even in patients with ejection fraction ≤35% in intermuscular implants, failure at ≤40 J being more common in the setting of a subcutaneous device placement.

Based on present results, larger studies are warranted to investigate whether lower defibrillation energies can reliably terminate VF in S‐ICD indications recipients, once an optimized implant is achieved: only two of the intermuscular recipients failed an attempt at ≤40 J in our series, though a well‐designed study with a strict methodology is required to prove the consistency of our observations on consecutive, unselected S‐ICD recipients. A 95% success rate at 45 J would enable to decrease the maximum S‐ICD output at 60 J, thereby increasing its longevity to state‐of‐the‐art transvenous ICDs,^14^ or could promote manufacturing of smaller devices meeting the clinical needs of pediatric as well as of small body habit patients.^15^ Indeed, although it has been recently shown that S‐ICD implantation is safe and effective in children and young adults,^15^ they would either benefit of a smaller can for acceptability or of long‐lasting S‐ICDs to avoid frequent replacements, that increase infection risk.^16^

The demonstration that the safety margin of currently adopted S‐ICD is frequently higher than what generally accepted for transvenous ICDs is also reassuring in situations where the defibrillation test is not performed. This occurred in 11% of our patients and in 19% of previous larger samples,^8^ for clinical reasons or for lack of VF inducibility. Most recent reports show that, despite a Class I recommendation, VF termination testing is declining in clinical practice due to physician preference.^13^ Definite data will derive from the ongoing randomized Trial of S‐ICD implantation with and without defibrillation testing (PRAETORIAN‐DFT), which aims to prove the safety of withholding defibrillation verification when implant optimization is based on the PRAETORIAN score.^17^

### 4.1 Limitations

This study is limited by its retrospective design, the small sample size, and the non‐uniform defibrillation testing protocol. In particular, we did not apply a step‐down or a small step‐up/reverse polarity testing in case of first attempt failure to accurately calculate the DFT. Nonetheless, the first‐attempt energy delivered was superior in subcutaneous S‐ICD recipients, which confirms the advantage of an intermuscular placement.

### 4.2 Conclusions

Our study shows a high rate of defibrillation success at low‐energy shock in consecutive S‐ICD recipients. Patients with reduced ejection fraction showed higher energy requirements, especially when placed subcutaneously. We believe that these observations are hypothesis‐generating for an accurate study to prove that a 60 J maximum output device may be as effective as an 80 J one, in a view to improve device longevity and/or suit small‐habit/pediatric patients.

## CONFLICT OF INTEREST

M.L. and S.V. are employees of Boston Scientific. M.B. reports educational activity and speaker bureau for Biotronik, Boston Scientific, Medtronic. M.Z. reports educational activity for Medtronic. The other authors report no conflict of interest.
